# Book Notices

**Published:** 1861-12

**Authors:** 


THE PRINCIPLES AND PRACTICE OF OBSTETRICS *By Gunning S. Bedford, A. M., M. D., Professor of Obstetrics, the Diseases of Women and Children, and Clinical Obstetrics, in the University of New York; Author of Clinical Lectures on the Diseases of Women and Children. Illustrated by four colored lithographic plates, and ninety-nine wood engravings. New York: Samuel S. & Wm. Wood,* 389 *Broadway,* 1861.

The above is the title of a new and original book, by an author already favorably known to the profession, whose means of observation in his peculiar department have been almost unlimited, and judging from the work before us, we are irresistibly led to the conclusion, that the author has well improved those opportunities, for the book presents internal evidence of his knowledge of the literature and a familiarity with the practical details of this department of the profession, which are embodied and systematically presented in this fine appearing volume.

So constant and rapid has been the march of improvement in the science and art of obstetrics for years past, that some means, besides fugitive contributions to medical journals, has become a frequent necessity to properly "post up" the profession upon the progressive and existing state of knowledge in this important branch of medical science. In proof of this, it is only necessary to refer to the large number of works of great value which, for the past few years, have followed each other in rapid succession, and that they were not uncalled for is evidenced by the repeated editions through which some of them have already passed.

The announcement of this truly American work by Prof. Bedford, cannot fail to excite great expectations in the minds of those who are familiar with the author's highly popular "Clinical Lectures on the Diseases of Women and Children," nor do we think such will be disappointed when they have carefully examined this new candidate for professional favor, or conclude that the author has sent his work out any too soon.

The arrangement of subjects is natural and philosophical, and they have been treated in a concise and perspicuous style, while the form of lectures which has been chosen will give to the work a peculiar charm for the student, without impairing its value or rendering it distasteful to the practitioner. The incidental allusions, sentiment and pathos occasionally thrown in, fulfill their intention, by illustrating principle, enforcing rules of practice, or interesting and rendering active the attention.

The author sets out from the usual starting point by giving in the first lecture a description of the bones of the pelvis, and impressing his class with the importance of the branch he teaches, and the necessity of being governed by "inflexible principle, namely: that the cardinal object of the accoucheur, when he crosses the threshold of the lying-in chamber, should be a conscientious exercise of his skill to mitigate as far as may be the sufferings of his patient and conduct her safely through the perils of her parturition."

In the second lecture, the divisions, articulations, measurements and inclined planes of the pelvis are clearly explained and illustrated by excellent cuts. Then follows a consideration of the fœtal head, its divisions and measurements, and this leads to a description of the mechanism of labor in cranial positions, in which the influence of the inclined planes is illustrated. The author shows his disposition to simplify the subject by adopting in his classification but four positions, agreeing in this with the most successful teachers and authors of the present time.

After devoting a lecture to deformities of the pelvis, the author passes to a description of the organs of generation and their physiology. The treatment of the subjects of menstruation and reproduction is admirable and fully up to the present state of physiological teaching upon these subjects. Here, as elsewhere through the work, the author makes just sufficient historical allusion to show how one hypothesis has displaced another, each succeeding one containing some new element of truth, until the profession has reached its present broad and comprehensive views of medical science.

The question, Is pregnancy a pathological condition? is then discussed. The constitutional sympathies excited in the system, and the changes in the blood might well lead to the inference that they must be the result of pathological changes; the conclusions reached, however, are expressed in the following terms: "That pregnancy is a modified condition of the system, but not a diseased condition; oftentimes complicated with disturbed action amounting to disease, which will require all the vigilance of the physician to arrest."

Considerable space is devoted to the consideration of the determining cause of labor, and, after reviewing and confuing the various theories, which have from time to time been advanced, to explain the initiatory of the process, the author advances his own views. It is not a little curious that upon this, as upon other mooted questions, it is not difficult for an individual to reach conclusions satisfactory to himself, without seeming to see how easily the evidence on which he relies can be disproved: eg. the ovarian theory—the maturity and disintegration of the placenta—the movements of the fætus, &c., all of which are demolished. Is not the author's explanation, which attributes the cause of this process to the "matured development of the muscular structure of the uterus," analogous to one which he has already disproved, viz: "the cessation of antagonism between the muscular fibres of the neck and body of the uterus?"

The author manifests a laudable desire to furnish an explanation for every change pecular to the pregnant female, as, for instance: "About the third month there is oftentimes quite a prominence of the hypogastric region, owing to the distension of the small intestines with flatus, which in a short time becomes measurably lessened, and thus at the fourth month the abdomen is frequently smaller than at the third." Considering it important to comprehend the cause of this difference, the following explanation is offered: "May it not be due in the first place to the irritation experienced by the ganglionic nerves of the uterus, and thus transmitted to the chylopoietic viscera, and secondly to a reflex influence occasioned by the physical changes going on in the uterus itself?" The reason given why this state of things does not continue during gestation is "because the digestive mechanism becomes in a short time accustomed to these combined influences." Would it not be as satisfactory to say, that as the uterus rises from its low position in the pelvis, that portion of the small intestines which occupied the sulcus between the uterus and bladder, pass over and back of the fundus uteri; and while making this transit they are brought in relation with the inferior portion of the abdominal wall for a short time, which gives this part the prominence referred to, and as they glide back the flatness re-appears?

The author reviews the doctrines which have by different persons been promulgated upon the management of placenta prævia, among others the *entire* separation of the placenta by Prof. Simpson — the *partial* separation recommended by Dr. Barnes. We cannot avoid the conviction that these views are likely to lead to confusion and grievous errors in practice, for it is undeniable that it would be a gratuitous assumption to suppose that every practitioner would fully comprehend the philosophy of these systems of practice, so as to be able to judiciously adopt the one or the other, as the emergency of the case might demand. For these reasons we endorse the teaching of our author, who recommends version at as early a period as practicable. It has not been our practice, however, to force the hand through the substance of the placenta in the operation.

Our limits will not permit us to gratify our inclination to follow the author, step by step, and note the peculiarities of this excellent work, for there is in it so much to commend and so little from which we could dissent, that this would afford us a real pleasure.

In taking leave of the subject for the present, we most cordially express our approbation of the work, for the scientific research and practical detail so evident on every page. Without making invidious comparisons, we believe that this work is destined to take the first place in the estimation of the profession, as an an exponent of the existing state of the science, and as such we commend it as worthy the confidence both of the student and practitioner.

In our opinion, the author deserves the thanks of the profession for having persevered in bringing out the work in the face of all the obstacles incident to so great an undertaking; and their gratitude for having produced a national work of such unequivocal value. M.

The work is for sale by S. C. Griggs & Co., 39 and 41 Lake street.

PLACENTA PRÆVIA. ITS HISTORY AND TREATMENT.—*By William Read, M. D., Member of the Massachusetts Medical Society; of the Boston Association: of the Society for Medical Improvement: of the Society for Medical Observation; of the Obstetrical Society of Boston; Lately Attending Physician at the Boston Lying-in Hospital. Philadelphia: J. B. Lippincott & Co.* 1861.

This monogrph of three hundred and forty pages will be read with interest by the profession, and any who may not be fully satisfied with the various exclusive and partial doctrines which have been advanced upon the management of this class of cases can consult this work with profit.

The author states, "that the work was begun for his own information, at a time when there was more doubt upon many points relating to this subject than at present. As the investigation proceeded, and materials accumulated, it was found that, although the labors of Prof. Simpson, Dr. Trask, and Dr. Barnes had thrown much light upon Placenta Prævia, there were, nevertheless, many questions upon which there might be a great difference of opinion."

A great amount of labor and patient research alone could enable the author to present the subject in so comprehensive and complete a form as he has done. It is indeed a valuable addition to the obstetric literature of the present day, and must meet the approbation of the profession.

The readers of the JOURNAL will thank us for inserting entire the general summary, which is presented in the following conclusions:

1st. The danger to the mother in Placenta Prævia *increases* as the period, at which the labor comes on, approaches the full term. A result rather to be expected from the increased capacity of the uterine vessels, as pregnancy advanced to its termination. It is therefore better to terminate the labor, after it has really begun, as soon as compatible with the safety of the patient, than to endeavor to conduct the pregnancy to the full term.

2d. The danger to the mother is less when the os uteri is completely covered, than when a portion only is involved in the attachment of the placenta; and least of all, where the attachment becomes nearly or quite central with reference to the os. Under these last conditions there is a strong probability, if the contractions are vigorous enough, that the placenta will be thrown off and expelled into the vagina, and the hemorrhage be checked.

3d. The *condition* of the mother, is a much more important element in making a prognosis of the case, than the amount of blood lost; some constitutions being very much less susceptible to the effect of depletion, and capable of sustaining a greater amount of hemorrhage without being unfavorably affected, than others. The condition of the mother then, should be most carefully watched, and the appearance of any symptoms indicating debility, or a tendency to collapse, should be the signal for the adoption of such remedies, or such a course as will the most speedily and safely insure the delivery of the child. And they should be put into effect without any delay, always bearing in mind the fact, that operations which are perfectly safe to the mother, when her vital power is comparatively undiminished and unimpaired, become almost certainly fatal, if performed when she has become exhausted by hemorrhage and suffering.

4th. In those cases where the pains are vigorous, and show a disposition to be permanent, (the head presenting, the os in good condition, and the strength not materially impaired,) *Rupturing the membranes*, by letting off the waters, and bringing the child's head down upon the os, will, in most instances, be enough to check the bleeding, and place the mother in a safe condition. When, however, a want of tonic power is manifested, or it is probable that resort must be had to forced delivery, the discharge of the waters in this way will only increase the difficulty of the operation, and the danger to the mother.

5th. The danger to the mother is materially increased by artificial delivery. But the same statistics which show this result, also make it evident that this increased fatality is owing *not so much to the operation itself*, as to the enfeebled and exhausted condition of the mother at the time; and that, with a favorable condition on the part of the mother, there is no more danger in resorting to it in Placenta Prævia, than in ordinary cases of difficult labor.

6th. The effect of artificial delivery to endanger the life of the mother in Placenta Prævia, being therefore almost directly proportionate to the degree of exhaustion under which she labors, it should be the aim of the practitioner to perform this operation, before such a state is reached; always bearing in mind the remark of Dr. Churchill, that "it is peculiar to midwifery operations that they form an ascending series, increasing in gravity from the simplest to the most severe—no two being equal; and therefore, in considering the suitability or practicability of any one, we do so with the knowledge that if the one we prefer, do not succeed, we must have recourse to another more severe and more dangerous."

7th. If from the progress of the case, or the conditions of the labor, a resort to artificial delivery must finally be had, it should not be delayed an instant beyond the time, when the dilatation, or dilatability of the os uteri, permits the introduction of the hand into the uterus: the danger to the mother from forced delivery, being directly proportionate to the degree of exhaustion under which she labors. (See art. 3d, 5th, 6th.)

8th. When from the rapidly failing condition of the mother, or the presence of any cause rendering artificial delivery impossible, a resort to the foregoing is forbidden, the placenta should be wholly separated from the uterus, and such remedies made use of (see Transfusion, p. 326,) as will recruit the strength of the mother, until reaction having been established, she can be delivered in whatever way may be deemed best.

9th. The tampon may be used advantageously in all those cases, where, with an amount of flooding sufficient to materially affect the constitution of the mother, the os uteri remains so rigid that it is impossible to perform artificial delivery. But, while under these circumstances, it is important to gain time for the dilatation of the os, and, at the same time, prevent the hemorrhage from too speedily exhausting the mother; under an opposite state of things a resort to the tampon, by inducing the temporizing policy, will often cause a loss of valuable time, and in this way make just the difference between a safe and a fatal issue. As the effect of this application is not only to check the hemorrhage, but also to excite labor-pains and dilatation of the os uteri, it is totally forbidden, in all cases where either or both of these results may not be desired.

10th. The effect of ergot being of a twofold nature, according to the condition of the system, (ecbolic or parturient when the nervous energy is undiminished, and stimulant when there is a want of this,) it should not be administered, when there is a probable necessity of terminating the labor by an oporation, unless at such an interval, that the effect of it is either exhausted, or will not come on until after the operation is finished, or the condition of the mother is such that it will act merely as a stimulant.

11th. In cases where the exhaustion is excessive, and version is the only alternative, after the feet have been brought down, the body of the child should be left undelivered, until the uterus has been roused to contract, and a firm condensation of its walls has been secured; or at least, it should be withdrawn so slowly, as to prevent the evil consequences which sometimes follow too sudden delivery.

---

MEDICAL JURISPRUDENCE.—*By Alfred Swaine Taylor, M. D., F. R. V. S. Fifth American, from the 7th and Revised London Edition. Edited, with additions, by Edward Hartshorn, M. D. Blanchard & Lea. Philadelphia,* 1861. *For sale by Wm. B. Keen, No.* 148 *Lake Street.*

---

The presentation to the profession of a new and improved edition of this well known and deservedly popular work, cannot be looked upon otherwise than as a subject of congratulation. The book has many merits. It is brief, it is comprehensive; it treats in a clear and satisfactory manner upon a large number of medico-legal subjects, the most interesting and important that can be presented to the attention of the physician, and the completeness of the work is enhansed, especially to the American reader, by the appropriate, though not very copious notes and references to recent American cases, by Dr. Hartshorn. The article on poisons is very perfect, treating of the definition of the term, of the different classes into which they are divided, as the irritant, corrosive, and neurotic; the circumstances which modify their effects, the signs to which they give rise in the subject, both living and dead; the indications which would justify the suspicion of poisoning, as well as some judicious cautions designed to prevent the practitioner from mistaking the manifestations of disease for those induced by toxical agencies; the mode of conducting the examination of cases of suspected poisoning; the peculiar symptoms induced by different poisonous substances; their proper tests, and the mode of their application. The article on infanticide, among other topics, considers the means of determining the uterine age of the child from an inspection of its body; the evidences that the child had lived, as furnished by the organs of respiration, by vital changes in the umbilical cord, the circulatory organs, &c., and closes with a review of the causes of death in new-born infants. The chapters on pregnancy and its signs; on delivery and its means of detection when concealed; on criminal abortion and the agencies by which induced; the signs both in the living and dead indicating that it has been committed, the laws relating to it, and the penalties prescribed as its punishment, convey to the mind clear ideas on the subjects therein discussed. The work contains articles of much interest on insanity, post-mortem changes and appearances, and numerous other subjects, of which time will scarce permit us to give even a synopsis.

Any physician need but place the work in his library, for its interest is sufficient to guarantee it a thorough perusal, and he will rise up from it better fitted to discharge the duties of his vocation. I.

